# Effects of bovine lipid extract surfactant administration in preterm infants treated for respiratory distress syndrome

**DOI:** 10.1002/hsr2.34

**Published:** 2018-03-24

**Authors:** Elizabeth Stockley, Ronald Valotaire, Michael Miller, Orlando da Silva

**Affiliations:** ^1^ Children's Hospital at London Health Sciences Centre Children's Health Research Institute London Ontario Canada

**Keywords:** BLES, FiO_2_, preterm, RDS

## Abstract

**Aim:**

To review the initial effectiveness of bovine lipid extract surfactant (BLES) for the treatment of respiratory distress syndrome in preterm infants.

**Methods and results:**

A retrospective review of data collected from infants born <37‐week gestation with respiratory distress syndrome treated with BLES between February 1, 2015 and March 1, 2016. Data were analyzed to determine the timing of initial dose, the length of time to wean the fraction of inspired oxygen (FiO_2_) concentration to 0.21 following initial dose, and the number of repeated doses given during hospital admission. Infants were subgrouped by gestational age stratum, 23^0^ to 27^6^ weeks (group 1), 28^0^ to 31^6^ weeks (group 2), and 32^0^ to 36^6^ weeks (group 3). Ninety‐eight infants received the surfactant during the study period. After applying exclusion criteria, 77 infants were analyzed. Mean (SD) gestational age was 28 (4) weeks, and mean (SD) birth weight was 1250 (602) g. Initial dose of BLES was given at a median (interquartile range) time of 29 (19‐43) minutes in group 1, 150 (20‐615) minutes in group 2, and 990 (53‐2025) minutes in group 3. Median (interquartile range) length of time to wean the FiO_2_ concentration to 0.21 was 14 (5‐56) minutes, 10 (5‐53) minutes, and 10 (5‐38) minutes in groups 1, 2, and 3, respectively. Ten infants required repeated doses.

**Conclusion:**

Given the rapid response of BLES in all the groups, careful monitoring of ventilator parameters is paramount to allow for rapid weaning and early extubation to avoid lung injury associated with mechanical ventilation.

## 1 INTRODUCTION

Surfactant replacement therapy reduces mortality and morbidity in preterm infants with respiratory distress syndrome (RDS).^1^, ^2^, ^3^, ^4^, ^5^ There are 2 types of surfactants: animal derived and protein‐free synthetic. Although both surfactant types are effective in the treatment and prevention of RDS, meta‐analyses have reported early improvement in ventilator support, improved mortality rates, and reduced rates of pneumothoraces with treatment with animal‐derived surfactants, compared with synthetic products.^6^ Animal‐derived surfactants differ in their phospholipids, surfactant proteins B and C, and plasmalogens composition, as well as their viscosity and volume adminstration.^7^, ^8^ While numerous randomized controlled trials have compared different animal‐derived preparations, it remains unclear whether significant differences in clinical outcomes exist among them.^5^, ^9^

Bovine lipid extract surfactant (BLES Biochemicals, London, Canada) is commonly used to treat RDS in premature infants in neonatal intensive care units (NICUs) across Canada, India, South Africa, Iran, Saudi Arabia, Bolivia, and Chile. It is a natural extract of bovine pulmonary surfactant,^9^, ^10^ containing 1% SP‐B and SP‐C and a phospholipid concentration of 27 mg/mL. Despite the popular use of BLES, there are limited published trials for its use in the treatment of RDS in preterm infants.^11^, ^12^ To our knowledge, there have not been any clinical studies specifically evaluating the immediate changes in fraction of inspired oxygen (FiO_2_) following administration of BLES. Understanding immediate response times of a specific surfactant is important, to allow for rapid weaning of ventilator parameters and early extubation onto noninvasive ventilator support, to ultimately avoid lung injury associated with ongoing mechanical ventilation. Our aim was to review the initial response of BLES by describing (1) the timing of the initial dose, (2) the length of time to wean the FiO_2_ concentration to 0.21 following initial dose only, and (3) the number of repeated doses given during hospital admission, for the treatment of RDS in premature infants.

## 2 METHODS

### 2.1 Design and participants

We performed a retrospective analysis of data of all preterm infants born between 23^0^‐ and 36^6^‐week gestation, admitted to the NICU at London Health Sciences Centre, London, Canada, with a diagnosis of RDS, and treated with BLES administration (5 mL/kg). The data were collected from a single‐study center, from February 1, 2015 until March 1, 2016. A diagnosis of RDS was clinical (tachypnea, intercostal retractions, nasal flaring, grunting, and an increasing FiO_2_ requirement to maintain oxygen saturation greater than 90%) and/or radiological.

The local evidence‐based practice guideline for the management of RDS was followed to determine which infants received BLES.^13^ The guideline includes all infants born less than 37‐week gestation at risk of developing RDS. Infants are resuscitated according to the Neonatal Resuscitation Program guidelines.^14^ Following resuscitation, each infant is assessed to determine which respiratory management strategy is the most appropriate.

1. No respiratory support: The infant does not require any supplemental oxygen or does not show any signs of increased work of breathing postresuscitation.
2. Nasal continuous positive airway pressure (NCPAP): If the infant shows signs of increased work of breathing and/or requires supplemental oxygen therapy postresuscitation, the infant is given a trial of NCPAP for a minimum of 10 minutes. If the FiO_2_ on NCPAP is <0.3 in the resuscitation room, the infant is transferred to the NICU on NCPAP. If the FiO_2_ increases to ≥0.4 on NCPAP in the NICU, the infant is considered for BLES administration.
3. Intubate surfactant extubate: If the FiO_2_ is ≥0.3 after a trial of NCPAP in the resuscitation room, the infant is eligible for intubate‐surfactant‐extubate method.
4. Surfactant and ongoing mechanical ventilation: If the infant is intubated and does not have an adequate respiratory effect and/or FiO_2_ is >0.3 following BLES administration and/or clinically unstable, the infant is not extubated following surfactant delivery.

Infants who are intubated during the resuscitation only, receive surfactant replacement therapy if a diagnosis of RDS is made. Repeated doses of BLES administration is considered if the FiO_2_ concentration is persistently >0.3 within the first 48 hours of life.

### 2.2 Data collection

The data were collected for each infant who received BLES either in the resuscitation room or in the NICU, by respiratory therapists. The following characteristics were collected from each infant: gestational age, birth weight (grams), clinical diagnosis, initial timing of BLES administration (minutes from birth), length of time to wean the FiO_2_ concentration to 0.21 following initial dose of surfactant replacement therapy, and number of repeated doses given during hospital admission. Only infants who were able to wean the FiO_2_ concentration to 0.21 after their initial dose were included in the main data analysis. Maternal characteristics were unavailable and were not collected.

To assess the effect of gestational age, infants were subgrouped by gestational age stratum: 23^0^‐ to 27^6^‐week gestation (extreme preterm: group 1), 28^0^‐ to 31^6^‐week gestation (very preterm: group 2), and 32^0^‐ to 36^6^‐week gestation (group 3). Infants born ≥37^0^‐week gestation who received BLES therapy were excluded from the study.

### 2.3 Statistical analysis

The statistical analysis was performed using SPSS v.24 (IBM Corp, Armonk, New York). The data were presented as mean ± standard deviation (SD), median ± interquartile range (IQR), and number of infants (%). Comparisons between gestational age subgroups were evaluated using Kruskal‐Wallis test. Statistical significance was defined as a *P* value < .05.

The study was approved by the Research Ethics Board at Western University, London, Ontario, Canada. Individual signed consent is not required to be obtained from patients/guardians for retrospective data/chart review, according to the Research Ethics Board guidelines. The data were extracted anonymously, with no identifiers. All parents are made aware on admission that our institution is a teaching/academic hospital and that their infant's data may be used for research.

## 3 RESULTS

**Figure 1:**
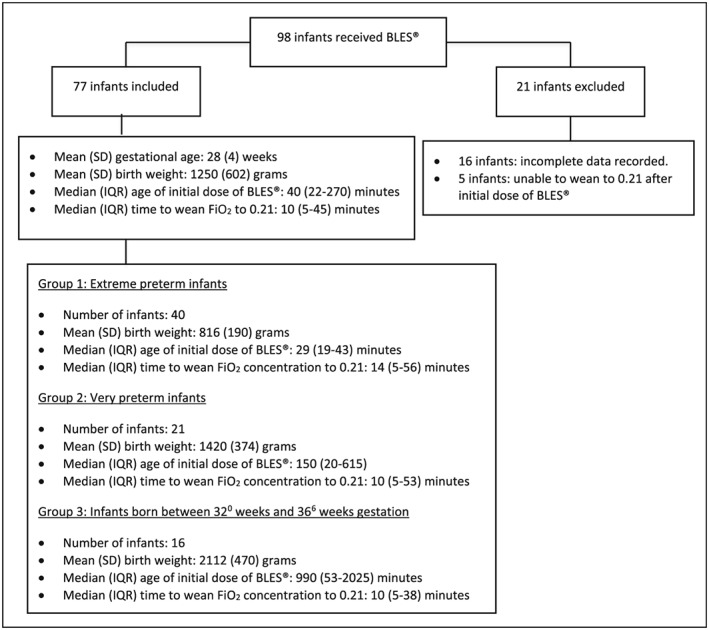
Flow diagram of results. BLES, bovine lipid extract surfactant; IQR, interquartile range; SD, standard deviation

Ninety‐eight infants born between 23^0^‐ and 36^6^‐week gestation with a diagnosis of RDS were treated with BLES during the study period (Figure 1). Twenty‐one infants were excluded in the initial analysis. Sixteen infants had incomplete data collected. Five infants had ongoing oxygen requirements and were unable to wean the FiO_2_ concentration to 0.21 following their initial dose of BLES; their mean (SD) gestational age was 28 (3) weeks, and the mean (SD) birth weight was 1342 (425) g.

Data from 77 infants were analyzed. Mean (SD) gestational age was 28 (4) weeks, and the mean (SD) birth weight was 1250 (602) g. The initial dose of BLES was given at a median (IQR) time of 40 (22‐270) minutes after birth, and the median (IQR) length of time to wean the FiO_2_ concentration to 0.21 was 10 (5‐45) minutes in all infants.

**Figure 2:**
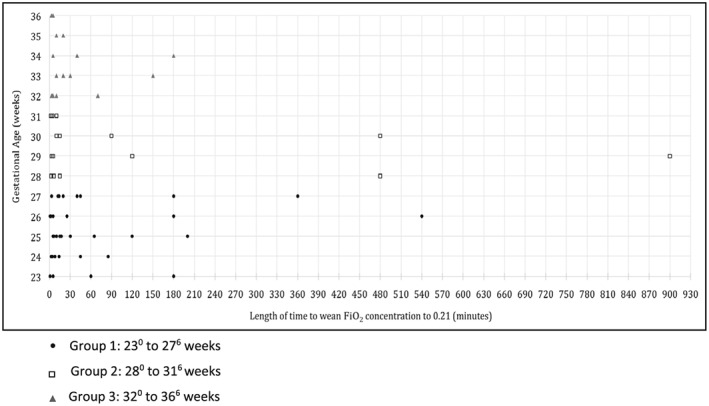
Response time of bovine lipid extract surfactant in all preterm infants

Infants were subgrouped by 3 gestational age stratum. This was based on a hypothesis that the smaller premature infants may show a different response to surfactant compared with the larger, more mature infants. Forty infants (52%) were in the extreme preterm group, mean (SD) birth weight 816 (190) g; 21 (27%) were in the very preterm group, mean (SD) birth weight 1420 (374) g; and 16 (21%) infants were born ≥32^0^‐week gestation, mean (SD) birth weight 2112 (470) g. A significant difference was observed in the timing of the initial dose of BLES between each group. In the extreme preterm group, the initial dose of BLES was given at a median (IQR) time of 29 (19‐43) minutes after birth, compared with 150 (20‐615) minutes in the very preterm group and 990 (53‐2025) minutes in infants born ≥32^0^‐week gestation (*P* < .001). However, no statistical significant difference was observed in the length of time to wean to 0.21 following initial dose, among the groups. In the extreme group, the median (IQR) length of time to wean the FiO_2_ concentration to 0.21 was 14 (5‐56) minutes in the extreme preterm group, 10 (5‐53) minutes in the very preterm group, and 10 (5‐38) minutes in infants born ≥32^0^‐week gestation (Figure 2).

Eighty‐eight percent (72/82) of preterm infants required only a single dose of BLES to wean the FiO_2_ concentration to 0.21. Ten infants required repeated doses. Among them, 5 infants initially weaned to 0.21 but subsequently developed an oxygen requirement and received further doses of BLES within 48 hours of birth. Four infants were in the extreme preterm group, and 1 infant was born ≥32^0^‐week gestation. Within the extreme preterm group, 3 infants received 2 repeated doses of BLES and 1 infant received 1 repeated BLES dose. The remaining 5 infants who required repeated doses had prolonged ongoing oxygen requirements following their initial dose of BLES and required 1 repeated dose within 48 hours of birth. Among them, 4 infants were in the extreme preterm group, and 1 infant was born ≥32^0^‐week gestation.

## 4 DISCUSSION

Our study is the first to describe the length of time to wean the FiO_2_ concentration to 0.21 following the initial dose of BLES administration in the treatment of RDS in preterm infants. All 3 groups, based on gestational age, showed a fast response to BLES, emphasizing the importance of careful monitoring of ventilator parameters to allow for rapid weaning and early extubation onto noninvasive ventilator support in infants of all gestations, to avoid pulmonary complications.

Extreme preterm infants received their initial dose of BLES significantly earlier in their RDS management compared with infants born ≥28^0^‐week gestation. This can be explained by the degree of RDS: Extreme preterm infants are more likely to have severe RDS at birth compared with infants with a higher gestational age. The local evidence‐based practice guideline may have also played a role, as criteria for BLES therapy varied according to the location of the infant (resuscitation room or NICU).

There are only 2 published trials comparing BLES with another animal‐derived surfactant. The initial study of Lam et al^11^ compared the clinical response by oxygenation indices of preterm infants with RDS, randomized to receive either beractant or BLES. Infants in the BLES group demonstrated a faster onset of action with a significantly lower oxygenation indices within the first 8 hours after initial dose and fewer days of mechanical ventilation. Lemyre et al^12^ compared the efficacy and safety of poractant alfa and BLES in infants <32‐week gestational age. They found no statistically significant difference in the proportion of infants alive and extubated within 48 hours, rates of bronchopulmonary dysplasia, mortality, or complications. However, duration of oxygen therapy was significantly reduced in infants who received poractant alfa.

Despite the limited data on BLES, there are numerous trials comparing the immediate response of different animal‐derived surfactants by means of changes in FiO_2_ at various time points.^8^, ^15^, ^16^, ^17^, ^18^, ^19^, ^20^ poractant alfa has been extensively studied and has been found to result in greater improvement in oxygenation and require fewer repeated doses when compared with Beractant.^8^, ^15^, ^16^, ^18^ However, there are no studies specifically evaluating the length of time to wean the FiO_2_ concentration to 0.21 following the administration of surfactant.

A retrospective review of our data found that 12% (10/82) of infants required repeated doses of BLES. Both Lam et al^11^and Lemyre et al^12^ reported redosing and found no significant difference between groups. However, they did observe higher rates than in our review of data. Thirty‐four percent (10/29) of infants required repeated doses of BLES compared with 58% (18/31) in the Beractant group,^11^ and 20% (9/45) of infants required repeated doses of BLES compared with 19% (8/42) in the poractant alfa group.^12^

Our review of data had some methodological limitations. First, this was a retrospective descriptive review, lacking a comparable control group. We also had a small sample size, which may result in lower statistical power to detect differences between groups. Finally, we did not analyze significant characteristics of the studied participants, such as sex, multiple gestation, perinatal asphyxia, Apgar scores, and maternal risk factors (preterm prolonged rupture of membrane, chorioamnionitis, mode of delivery, and prenatal steroids); these factors may have affected our results.

## 5 CONCLUSION

Extreme preterm infants received their initial dose of BLES earlier in their RDS management. Fast onset of action with rapid weaning of the FiO_2_ concentration to 0.21 was described in all 3 groups of preterm infants following the administration of BLES for the treatment of RDS. Careful monitoring of ventilator parameters following BLES administration is therefore paramount to allow for rapid weaning and early extubation onto noninvasive ventilator support, to prevent lung injury associated with mechanical ventilation.

## CONFLICT OF INTERESTS

BLES Biochemical Inc did not fund the study nor had any influence in the design, data collection, and interpretation of the results. The surfactant maker did not review or revised the manuscript submitted for publication.

## AUTHOR CONTRIBUTION

Conceptualization: Orlando da Silva, Elizabeth Stockley

Formal analysis: Michael R Miller

Data abstraction: Ron Valotaire, Elizabeth Stockley

Writing – original draft preparation: Elizabeth Stockley

Writing – review and editing: Orlando da Silva, Michael R Miller, Ron Valotaire, Elizabeth Stockley
